# Fluvoxamine for aripiprazole-associated akathisia in patients with schizophrenia: a potential role of sigma-1 receptors

**DOI:** 10.1186/1744-859X-9-11

**Published:** 2010-03-06

**Authors:** Tsutomu Furuse, Kenji Hashimoto

**Affiliations:** 1 Department of Psychiatry, Asahikawa Red Cross Hospital, Asahikawa, Japan; 2 Division of Clinical Neuroscience, Chiba University Center for Forensic Mental Health, Chiba, Japan

## Abstract

**Background:**

Second-generation antipsychotic drugs have been reported to cause fewer incidences of extrapyramidal side effects (EPSs) than typical antipsychotic drugs, but adverse events such as akathisia have been observed even with atypical antipsychotic drugs. Although understanding of the pathophysiology of akathisia remains limited, it seems that a complex interplay of several neurotransmitter systems might play a role in its pathophysiology. The endoplasmic reticulum protein sigma-1 receptors are shown to regulate a number of neurotransmitter systems in the brain.

**Methods:**

We report on two cases in which monotherapy of the selective serotonin reuptake inhibitor and sigma-1 receptor agonist fluvoxamine was effective in ameliorating the akathisia of patients with schizophrenia treated with the antipsychotic drug aripiprazole.

**Results:**

The global score on the Barnes Akathisia Scale in the two patients with schizophrenia treated with aripiprazole decreased after fluvoxamine monotherapy.

**Conclusion:**

Doctors may wish to consider fluvoxamine as an alternative approach in treating akathisia associated with antipsychotic drugs such as aripiprazole.

## Background

Second-generation antipsychotic drugs have been reported to cause fewer incidences of extrapyramidal side effects (EPSs) than typical antipsychotic drugs, but adverse events such as akathisia have been observed even with atypical antipsychotic drugs. Akathisia is one of the most common and distressing EPSs of antipsychotic drugs [1,2]. The development of akathisia can adversely affect patients' adherence to medication, and, as a consequence, have a negative impact on long-term treatment outcomes in patients with schizophrenia [3,4]. Although therapeutic drugs (for example, β-adrenergic blockers, benzodiazepines, and anticholinergic drugs) have been used in the treatment of akathisia, they show only a moderate efficacy, and a substantial proportion of patients fail to respond to treatment. In contrast, understanding of the pathophysiology of akathisia remains limited. Given the clinical profile of akathisia, it seems that a complex interplay of several neurotransmitter systems (for example, dopamine, acetylcholine, norepinephrine, serotonin, γ-aminobutyric acid (GABA), and neuropeptides) underlies its complex pathophysiology [1,2].

The endoplasmic reticulum protein sigma-1 receptors play a key role in Ca^2+ ^signalling and cell survival, and have been shown to regulate a number of neurotransmitter systems in the central nervous system [5-8]. A recent study identified the sigma-1 receptors as possessing innate biological activity as a molecular chaperone, activity that can be activated/inactivated by synthetic compounds that bind to sigma-1 receptors [9,10]. Furthermore, sigma-1 receptors play important roles in the Ca^2+ ^signalling and bioenergetics within the cell [8-10]. The selective serotonin reuptake inhibitor (SSRI) fluvoxamine is a very potent agonist on sigma-1 receptors [11,12]. A study using a selective sigma-1 receptor agonist [^11^C]SA4503 and positron emission tomography demonstrated that fluvoxamine binds to sigma-1 receptors in living human brain at therapeutic doses, suggesting that sigma-1 receptors might play a role in the mechanism of action of fluvoxamine [13].

Given the important role of sigma-1 receptors in the regulation of neurotransmitter systems, we hypothesised that fluvoxamine may be effective in the treatment of akathisia associated with antipsychotic treatment. Aripiprazole is an antipsychotic drug that acts as a partial agonist at dopamine D_2 _receptors and serotonin 5-hydroxytryptamine (5-HT)_1A _receptors, and an antagonist at 5-HT_2A _receptors. The Schizophrenia Trial of Aripiprazole (STAR) study demonstrated a relatively higher incidence of akathisia with aripiprazole compared with placebo or other antipsychotic drugs (olanzapine, quetiapine, or risperidone)[14]. Here, we report two cases in which fluvoxamine was effective in treating aripiprazole-induced akathisia in patients with schizophrenia. Written informed consents were obtained from the all patients in this case report.

## Case reports

### Case 1

The patient was a 24-year-old woman who met the Diagnostic and Statistical Manual of Mental Disorders, fourth edition (DSM-IV) criteria for schizophrenia. Treatment with aripiprazol (12 mg) was initiated; 2 days later, the patient complained of leg restlessness. Her global score on the Barnes Akathisia Scale [15] was 3 ('moderate akathisia'). Fluvoxamine (50 mg, twice a day) was administered. Substantial relief of akathisia was noted on day 7 of fluvoxamine treatment. The dose of aripiprazole was increased to 24 mg, since she still had persecutory delusions and auditory hallucinations. Fluvoxamine (50 mg) continued to be administered. After 3 weeks, she had no recurrence of the akathisia.

### Case 2

The patient was a 41-year-old man who met the DSM-IV criteria for schizophrenia. Because of quetiapine-induced body weight gain, the antipsychotic drug was changed to aripiprazole (6 mg). He showed signs of akathisia after the dose of aripiprazole was increased to 12 mg. His global score on the Barnes Akathisia Scale was 3. Administration of fluvoxamine (50 mg, twice a day) rapidly improved the akathisia. He showed no signs of akathisia after the dose of aripiprazole was increased to 24 mg, his body weight decreased, and his mental status was stable.

## Discussion

To our knowledge, this is the first report demonstrating that fluvoxamine is effective in the treatment of aripiprazole-induced akathisia of patients with schizophrenia. Furthermore, we have experienced that fluvoxamine is also effective in the treatment of other antipsychotic-induced akathisia in patients with schizophrenia (data not shown). Nonetheless, a randomised double-blind, placebo-controlled study of fluvoxamine will be needed to confirm its efficacy for the treatment of this syndrome. From these case studies, it is unclear whether sigma-1 receptor agonism appears to be irrelevant to the anti-akathitic action of fluvoxamine. In order to confirm the role of sigma-1 receptors in the treatment of akathisia, a randomised double-blind, placebo-controlled study of the selective sigma-1 receptor agonist (for example, cutamesine (SA4503)) in patients with antipsychotic-induced akathisia would be also of interest.

Akathisia is a neurological side effect of antipsychotic medications, which are used to treat various psychiatric disorders such as schizophrenia and bipolar disorders [1,2,4]. It seems that akathisia is simply a dopamine D_2 _receptor blockade [1] although the precise mechanisms underlying antipsychotic drugs-induced akathisia are currently unclear. A number of neurotransmitter systems play a role in the complex pathophysiology of akathisia [1,2]. At present, it is unclear whether sigma-1 receptor agonism is involved in the mechanisms of anti-akathitic action of fluvoxamine. Considering the important role of sigma-1 receptors in the regulation of a number of neurotransmitter systems [5-8], it is likely that indirect modulation of several neurotransmitter systems by sigma-1 receptor agonist may be involved in the mechanisms of this drug although a further detailed study will be necessary.

## Conclusions

These two cases suggest that fluvoxamine may serve as an alternative option in the treatment of antipsychotic-induced akathisia in patients with schizophrenia. More detailed randomised, double-blind studies of fluvoxamine using larger samples should be performed to clarify the role of sigma-1 receptors in the efficacy of fluvoxamine for akathisia.

## Competing interests

The authors declare that they have no competing interests.

## Authors' contributions

TF contributed to the clinical and rating evaluations during the follow-up periods. KH conceived of the study and participated in its study and coordination. All authors read and approved the final manuscript.
